# Relationship Between Perceived Stress, Midwife Support and Exclusive Breastfeeding Among Polish Mothers

**DOI:** 10.3390/nu17091573

**Published:** 2025-05-02

**Authors:** Agnieszka Czerwińska-Osipiak, Anna Weronika Szablewska, Wiktoria Karasek, Aleksandra Krawczyk, Krzysztof Jurek

**Affiliations:** 1 Department of Obstetric and Gynaecological Nursing, Institute of Nursing and Midwifery, Medical University of Gdańsk, Sklodowskiej Curie 3A, 80-210 Gdańsk, Poland; wiktoriakarasek@gumed.edu.pl (W.K.); alekra@gumed.edu.pl (A.K.); 2 Institute of Sociological Sciences, Faculty of Social Sciences, John Paul II Catholic University of Lublin, Al. Racławickie 14, 20-950 Lublin, Poland; krzysztof.jurek@kul.pl

**Keywords:** breastfeeding, lactation, infant, newborn, maternal–child health services, maternal stress, perceived stress, midwife’s role

## Abstract

Background/Objectives: Breastfeeding is a cornerstone of infant nutrition, promoting optimal development and health benefits for both mother and child. Despite high initiation rates in Poland (97%), exclusive breastfeeding (EBF) drops sharply, reaching only 4% by six months postpartum. The aim of this study is to identify factors associated with breastfeeding practices and barriers to exclusive breastfeeding (EBF) among Polish women during the postpartum period, with particular emphasis on the role of medical personnel support and maternal stress levels. Methods: A cross-sectional observational study, adhering to STROBE guidelines, was conducted from January to May 2023. The study included 1092 Polish women, surveyed using the Computer-Assisted Web Interview (CAWI) methodology. The women exclusively breastfeeding accounted for 79% (*n* = 863) of the study group. The remaining women supplemented their child with modified milk (*n* = 229; 21%). Statistical analyses were performed using IBM SPSS Statistics (Version 26.0), and logistic regression to assess associations between variables and breastfeeding outcomes. Results: Logistic regression analysis indicated that in the women experiencing low or medium stress, none of the analysed support factors significantly influenced the likelihood of exclusive breastfeeding discontinuation. However, women experiencing high stress, receiving counselling for effective breastfeeding (OR = 0.467; 95% CI: 0.232–0.941; *p* = 0.033) and assistance with proper breastfeeding (OR = 0.424; 95% CI: 0.220–0.819; *p* = 0.011) were associated with a lower likelihood of introducing formula feeding. The main reported reasons for early breastfeeding cessation included breast health issues, mental exhaustion, lack of medical support and infant-related difficulties. Conclusions: The findings allow us to underscore the urgent need for targeted interventions to improve breastfeeding rates in Poland. New evidence indicates that women experiencing higher levels of stress require increased support from medical personnel in order to breastfeed exclusively.

## 1. Introduction

Breastfeeding remains a crucial element in the health and well-being of both mothers and their newborns, fostering an intimate bond and providing essential nutrients for optimal growth and development [1]. The introduction of formula for infants into the global and Polish markets has led to evolving recommendations regarding infant feeding practices. Along with the increase in access to ready-made formulas, shifts in attitudes towards breastfeeding have been observed, both among women and healthcare professionals [2,3]. This trend continues to be evident today. Despite the well-documented and scientifically supported benefits of breastfeeding for both the infant and mother, a significant share of women still opt for formula feeding, thereby denying infants the unique and irreplaceable components present in breast milk [4,5].

Breastfeeding offers essential immunological and developmental advantages for infants. It significantly reduces the risk of respiratory tract infections, otitis media, gastrointestinal infections, asthma, type 1 diabetes and childhood obesity. In addition, breastfeeding supports optimal brain development and has been linked to improved cognitive outcomes later in life [6,7]. For mothers, breastfeeding is associated with a lower risk of developing breast and ovarian cancers, type 2 diabetes and cardiovascular disease. It also promotes uterine involution, helps with postpartum weight loss and delays the return of menstruation, acting as a natural contraceptive in the early postpartum period. Importantly, the release of oxytocin during breastfeeding may reduce maternal stress and enhance emotional bonding with the child [8].

The World Health Organization (WHO) defines exclusive breastfeeding (EBF) as the gold standard in infant nutrition, offering numerous benefits for both the child and mother [9]. However, the lack of current data on the number of newborns and infants exclusively breastfed, as well as the duration of breastfeeding among the Polish population, hinders accurate estimation regarding the national scope of exclusive breastfeeding until the infant is six months old [4]. Poland stands out for its relatively high percentage of women initiating breastfeeding compared to other European countries and the world [10,11]. The latest available population-based data are from 2017 and indicate that nearly all women in Poland initiate breastfeeding at birth (97%) [12]. However, the proportion of breast milk in an infant’s diet decreases significantly over the following months, with 43.5% of infants being exclusively breastfed by the second month, and only 4% of Polish children being exclusively breastfed at six months [12,13]. It is important to note that global rates of exclusive breastfeeding have increased in recent years; however, significant disparities persist between countries [14].

In a 2019 report from 11 European countries (Belgium, Croatia, Denmark, Germany, Ireland, Italy, the Netherlands, Norway, Spain, Sweden, and Switzerland) significant variation is highlighted with regard to breastfeeding rates, data collection methodologies and the mechanisms in place for supporting, protecting and promoting breastfeeding. Immediately after birth, the share of newborns receiving breast milk ranges from 56% to 98% across these countries [15]. However, by the age of six months, the percentage of infants still breastfed declines to between 38% and 71%, while the proportion of infants exclusively breastfed ranges from 13% to 39% [4]. These findings underscore the researchers’ conclusion that national governments must commit to evidence-based strategies for monitoring and promoting breastfeeding. This includes providing financial and policy support aimed at improving breastfeeding rates across Europe [16]. Furthermore, enhanced cooperation between countries is essential to establish a sustainable platform for information sharing and best practice dissemination.

The aforementioned figures do not align with one of the global nutrition targets for 2025, which aims to increase the rate of exclusive breastfeeding during the first six months to at least 50% [16]. The availability of timely and accurate data on the prevalence of breastfeeding among women across different countries is essential for monitoring progress in breastfeeding promotion efforts and identifying key areas that require intervention.

Breastfeeding practices are shaped by a complex interplay of sociodemographic factors, including maternal age, education level, socioeconomic status and cultural norms. Supportive factors, such as access to lactation counselling, workplace accommodations and strong family or community support, have been associated with higher breastfeeding initiation and continuation rates. Breastfeeding should not be seen as the sole responsibility of the mother. In numerous research reviews, it has been shown that effective improvements in breastfeeding rates at the population level require a concerted effort by society as a whole [3]. Conversely, barriers, such as insufficient maternity leave policies, limited public breastfeeding spaces and societal stigma, can hinder breastfeeding practices [17,18]. In Poland, as in other countries, these factors contribute to disparities in breastfeeding rates, necessitating a comprehensive understanding of the challenges, and an enabling of the conditions required, to inform evidence-based public health interventions aimed at promoting breastfeeding across diverse populations.

It is well documented that pregnancy and the postpartum period are associated with numerous psychosocial stressors, which are known to be linked to early negative health outcomes in infants. A systematic review of 48 studies found that postpartum depression, in particular, is a predictor of breastfeeding cessation [19]. However, there is a lack of updated systematic reviews focusing on the study of stress and breastfeeding during the postpartum period. Becoming a mother can be particularly challenging, as it involves adopting a new role and new responsibilities, thus becoming a source of stress. Maternal stress and limited social support may be associated with early cessation of EBF. Current evidence suggests that simultaneous screening for breastfeeding difficulties and maternal stress are important warning signs that may indicate complications even before a mother tests positive for postpartum depression (PPD) [20].

In addition to psychosocial stressors and postpartum depression, several other factors have been identified as significant contributors to early cessation of exclusive breastfeeding. caesarean section deliveries, for instance, are associated with a higher risk of delayed initiation and early discontinuation of breastfeeding. In a systematic review, it was indicated that the odds of mothers who underwent caesarean sections were 1.69 times more likely to cease exclusive breastfeeding than those who had vaginal deliveries [21] Maternal obesity is another critical factor; an elevated pre-pregnancy body mass index (BMI) has been linked to reduced breastfeeding initiation rates and shorter breastfeeding duration [22]. Furthermore, socioeconomic status plays a pivotal role; lower socioeconomic status has been associated with decreased breastfeeding initiation and duration [23]. These factors, alongside limited breastfeeding education and support, underscore the multifaceted nature of breastfeeding challenges.

Understanding these factors is crucial, as the long-term benefits of breastfeeding for children are well established, particularly with regard to its role in preventing obesity and type 2 diabetes. With the prevalence of childhood obesity on the rise globally, as highlighted in recent reports [24,25], breastfeeding emerges as a key preventive measure to support healthier weight trajectories and reduce the risk of chronic conditions later in life.

Despite the growing body of international studies in the literature on factors influencing breastfeeding, there remains a lack of comprehensive research focused specifically on the Polish population. The aim of this study is to explore factors related to breastfeeding outcomes in the Polish context. In Poland, the most current available data on breastfeeding collected by the Central Statistical Office of Poland (GUS) were obtained in 2014 and 2019 as part of the European Health Interview Survey (EHIS) [26]. This study included information on the percentage of infants breastfed in the first months of life. However, these data were not collected regularly and do not provide detailed insights into the duration and methods of infant feeding in Poland. Since then, GUS has not conducted any large-scale, population-based studies on breastfeeding. This lack of consistent national monitoring limits the ability to develop evidence-based breastfeeding support policies. Current data primarily come from scientific research and individual reports such as those from the Institute of Mother and Child (IMiD) or non-governmental organisations.

In this cross-sectional observational study, the gap in the current literature is addressed by examining the interplay between maternal stress levels, the support provided by medical personnel and lactation success. To the authors’ knowledge, in no prior large-scale study in Poland have these psychosocial and professional factors been assessed within the context of breastfeeding outcomes. The findings may have significant implications for clinical practice, offering insights that could guide the development of targeted interventions to enhance breastfeeding support for mothers. By identifying key determinants of successful breastfeeding, including the impact of maternal stress and the crucial role of midwife support, this research is aimed at improving health outcomes for both mothers and their children, emphasising the promotion of breastfeeding as a public health priority.

## 2. Materials and Methods

### 2.1. Study Design and Setting

The present observational, cross-sectional study was designed to assess breastfeeding practices among Polish women in the postpartum period (up to six months postpartum). Variables were selected that informed about practices supporting breastfeeding. They were provided by medical personnel and allowed to take maternal stress levels into account. These variables included aspects related to exclusive breastfeeding as well as a subgroup of women who supplemented their infants despite recommendations for EBF. The study adhered to STROBE guidelines for cross-sectional studies [27]. All procedures were performed in accordance with the principles outlined in the 1964 Declaration of Helsinki, created by the World Medical Association (WMA) and were approved by the Bioethics Commission of the Gdańsk Medical University, No. NKBBN/159/2023, for studies involving humans.

The study sample included 1092 Polish women; women exclusively breastfeeding accounted for 79% (*n* = 863) of the study group. The remaining women supplemented their child with modified milk (*n* = 229; 21%). Data were collected between January 2023 and May 2023 using the Computer-Assisted Web Interview (CAWI) methodology, an online survey administered via a dedicated link. This research method allowed for the collection of data from a wide-ranging and diverse group of participants across Poland.

Participants were recruited from online platforms and social media groups dedicated to motherhood, which taken together, comprised approximately 14,000 members from across Poland. To minimise selection bias, private and local groups were excluded, as were groups specifically focused on breastfeeding. This was done to ensure the inclusion of participants with varying feeding preferences and experiences. Furthermore, additional efforts were made to reach mothers who planned to use formula feeding, thus providing a more comprehensive perspective on infant feeding practices. All participants were informed of the study’s objectives; their written, informed consent was obtained before completing the survey. To enhance data reliability, the questionnaire included control questions to identify potential inconsistencies in responses; duplicate submissions from the same IP address were removed.

The online survey was carried out via a questionnaire link that was shared through social media platforms. The survey included an introductory section, informing respondents of the study’s objectives, providing instructions for completing the questionnaire and emphasising the voluntary nature of participation, with the option to withdraw at any point without providing justification.

Additionally, participants were given the opportunity to provide open-ended comments at the end of the survey, allowing for qualitative insights that complemented the quantitative data.

### 2.2. Participants and Inclusion/Exclusion Criteria

All participants were informed about the study’s objectives and provided their voluntary consent to participate by indicating their agreement within the questionnaire.

#### 2.2.1. Sample Size Calculation

The sample size calculation was based on statistical data regarding births in Poland, as reported by the Central Statistical Office (GUS). According to the Demographic Yearbook 2023 [28] (data as of December 2022), the female population aged 18–49 years totalled 8,670,951. In the same year, 305,132 live births were recorded. Assuming a 5% significance level and an acceptable error margin (e) of 3%, the minimum required sample size was calculated to be 1064 respondents. The final sample comprised 1092 respondents, which exceeds the minimum threshold, confirming its adequacy for statistical analysis.

#### 2.2.2. Recruitment and Initial Sample

The study initially involved 1203 women who voluntarily completed the survey questionnaire. However, after applying the inclusion and exclusion criteria, the final sample consisted of 1092 respondents with correctly completed questionnaires.

#### 2.2.3. Inclusion Criteria

The primary inclusion criterion was the child’s age, which was required to be no more than six months at the time of the survey. This specific age range was chosen based on the recommendations of global organisations such as the World Health Organization (WHO) and the European Society for Paediatric Gastroenterology, Hepatology and Nutrition (ESPGHAN) [9]. These guidelines advocate for exclusive breastfeeding during the first six months of life. Beyond this period, mothers typically begin introducing complementary foods into their infants’ diets, which could influence breastfeeding frequency and potentially introduce bias into the assessment of breastfeeding practices.

#### 2.2.4. Exclusion Criteria

Participants were excluded from the study for two main reasons. First, respondents whose child was older than six months at the time of survey completion were excluded (*n* = 101). Second, respondents who provided internally inconsistent or contradictory answers were also excluded. These inconsistencies included: reporting cohabitation with a partner and the child’s father while simultaneously identifying as a single mother (*n* = 5); stating that they did not utilise post-natal midwife visits while also describing breastfeeding support received during those visits (*n* = 5); reporting no formula supplementation but indicating a non-zero number of formula feeds; and declaring breastfeeding while either reporting zero breastfeeding sessions or specifying an exclusive breastfeeding duration longer than 26 weeks.

#### 2.2.5. Final Sample Size

Based on the inclusion and exclusion criteria, 111 participants were excluded from the initial sample (*n* = 111). The final dataset included responses from 1092 women, representing a valid and statistically sufficient sample for the study’s objectives.

**Figure 1:**
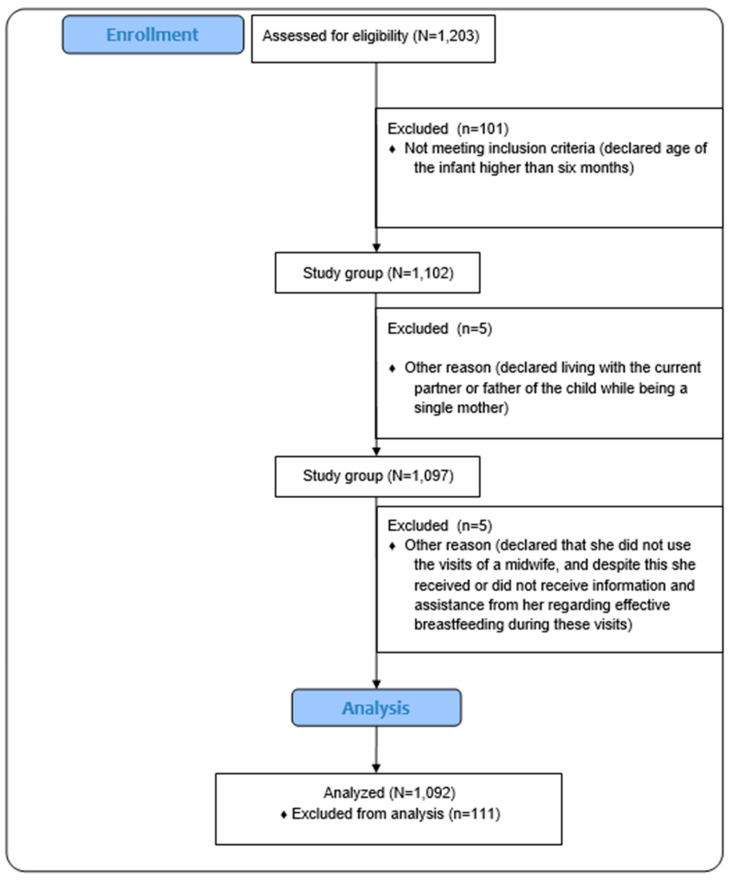
The flow of participants through the study.

The participants’ flow through the study is presented in Figure 1.

### 2.3. Data Collection Tools

The data for this study were collected using a structured, author-developed questionnaire. The questionnaire was designed by a research team of 10 experts—midwives holding the title of Certified Lactation Consultant—having extensive experience working with breastfeeding mothers. The questionnaire was designed based on their practical knowledge and experience, incorporating current recommendations and guidelines related to breastfeeding. It is a tool grounded in solid professional expertise, with its structure and questions tailored to the actual needs of breastfeeding mothers.

To ensure the validity and accuracy of responses, the questionnaire items were independently assessed by experts using a five-point scale. Items that received a minimum score of three points were included in the final version of the tool, ensuring that the selected questions met the required standards for relevance and clarity.

The survey questionnaire consisted of three distinct sections and included a mix of single- and multiple-choice questions. These sections were designed to comprehensively capture the following aspects of demographic and social data presented below.

#### 2.3.1. Demographic and Social Data

This section comprises a collection of information on the participants’ age, education level, marital status, place of residence and employment status, providing a background for analysing breastfeeding practices across various demographic profiles.

#### 2.3.2. Infant Feeding Practices

The Table 1 below summarizes the infant feeding variables included in the analysis. It presents information on feeding methods, the duration of exclusive breastfeeding, and the frequency of both breastfeeding and formula feeding.

#### 2.3.3. Maternal Opinions and Perceptions

This section was dedicated to mothers’ opinions on breastfeeding, including:

- perceived benefits of breastfeeding (e.g., health benefits for the baby, bonding with the mother, convenience);
- challenges associated with breastfeeding.

#### 2.3.4. Support and Care from Medical Professionals

Participants were asked about the type and quality of support received from healthcare professionals, such as midwives and lactation consultants, in accordance with Poland’s national standards for perinatal care [29].

#### 2.3.5. Perceived Stress Scale (PSS-10)

The perceived stress intensity among participants was evaluated using the Polish version of the Perceived Stress Scale (PSS-10) [30], a validated tool widely used in psychological research. The PSS-10 comprises 10 questions designed to measure how stressful participants perceive their life situations. Responses are scored on a five-point Likert scale (0 = never, 4 = very often), with higher scores indicating greater perceived stress.

Scores ranging from 0–13 would be considered low stress (1–4 sten). Scores between 14–19 would be considered moderate stress (5–6 sten). Scores ranging from 20–40 would be considered high perceived stress (7–10 sten) [31]. The level of Cronbach’s alpha was 0.90 in this study.

### 2.4. Variables

- The dependent variable (DV) was breastfeeding, defined as the actual practice of breastfeeding, including both initiation and continuation. Breastfeeding was categorised as a binary variable: (1) exclusive breastfeeding, meaning the infant received only breast milk without supplementation, and (2) non-exclusive breastfeeding, which included partial breastfeeding (breast milk combined with formula or solid foods) or no breastfeeding at all;
- Breastfeeding support factors: counselling for effective breastfeeding (yes/no), assistance with breastfeeding (yes/no), use of home midwife visits (yes/no), receiving information and assistance from a midwife (yes/no) and use of formula feeding (yes/no);
- Psychological factors: stress levels measured using the Perceived Stress Scale (PSS-10), with higher scores indicating greater perceived stress. This variable was used for determining two groups: women with low or average stress and women with high stress. Scores ranging from 0–13 would be considered low stress. Scores ranging from 14–19 would be assumed as moderate stress. Scores ranging from 20–40 would be viewed as high perceived stress.

### 2.5. Statistical Analysis

Data analysis was conducted using IBM SPSS Statistics (version 26.0; SPSS Inc., Chicago, IL, USA), ensuring robust and reliable statistical assessment. To identify factors related to the discontinuation of exclusive breastfeeding, logistic regression analysis was performed using the enter method, with exclusive breastfeeding (1) vs. non-exclusive breastfeeding, complementary feeding of the baby with formula (0). Model fit was evaluated via relevant regression statistics. Odds ratios (ORs) with 95% confidence intervals (CIs) were calculated for all independent variables. All statistical tests were two-tailed, with a significance level set at alpha = 0.05.

## 3. Results

### Characteristics of Study Group

A total of 1092 women participated in the study. The women exclusively breastfeeding accounted for 79% (*n* = 863) of the study group. The remaining women supplemented their child with modified milk (*n* = 229; 21%). The mean age of the participants was 30.63 years (SD = 4.04), ranging from 18 to 44 years. The demographic and social data of the women participating in the study are presented in Table 2.

In Table 3, the results are presented for the logistic regression analysis identifying factors associated with the discontinuation of exclusive breastfeeding in a group of women who experienced low or moderate stress. Logistic regression analysis was used to examine the relationship between breastfeeding support factors, including: counselling for effective breastfeeding (yes/no); assistance with breastfeeding (yes/no); use of home midwife visits (yes/no); receiving information and assistance from a midwife (yes/no); and complementary feeding of the baby with formula (yes/no). The model did not show satisfactory statistical properties: χ^2^ = 5.936, *p* = 0.204. None of the examined variables demonstrated a statistically significant effect on the outcome. Concealing for effective breastfeeding was not significantly associated with the discontinuation of exclusive breastfeeding, either in the crude analysis (Crude OR = 0.852; 95% CI: 0.573–1.265; *p* = 0.427) or after adjustment for potential confounders (AOR = 1.085; 95% CI: 0.595–1.976; *p* = 0.790). Similarly, assistance in properly breastfeeding showed no significant association (Crude OR = 0.731; 95% CI: 0.494–1.040; *p* = 0.119; AOR = 0.760; 95% CI: 0.418–1.382; *p* = 0.369). The use of midwife visits at home did not significantly influence the outcome either (Crude OR = 1.081; 95% CI: 0.466–2.509; *p* = 0.856; AOR = 1.375; 95% CI: 0.567–3.333; *p* = 0.481). Finally, receiving information and assistance from a midwife was not significantly associated with the discontinuation of exclusive breastfeeding, although the adjusted odds ratio (Crude OR = 0.734; 95% CI: 0.494–1.090; *p* = 0.119; AOR = 0.654; 95% CI: 0.421–1.017; *p* = 0.059) suggested a trend towards significance.

In Table 4, the results are presented of logistic regression analysis identifying factors associated with exclusive breastfeeding in a group of women who experienced high stress. The model demonstrated satisfactory statistical properties: χ^2^ = 32.780, *p* < 0.001, with Nagelkerke’s R^2^ = 0.116. Model validity was further assessed using the Hosmer-Lemeshow test, yielding χ^2^ = 5.624, *p* = 0.345, indicating a good fit. The logistic regression model accurately predicted group membership in 76.8% of cases.

Concealing for effective breastfeeding was significantly associated with the discontinuation of exclusive breastfeeding. In crude analysis, mothers who concealed themselves, had significantly lower odds of the outcome compared to those who did not (Crude OR = 0.272; 95% CI: 0.157–0.471; *p* < 0.001). This association remained significant after adjusting for potential confounders (AOR = 0.467; 95% CI: 0.232–0.941; *p* = 0.033). Similarly, assistance in properly breastfeeding was also significantly associated with the outcome. Mothers who received assistance demonstrated lower odds regarding exclusive breastfeeding discontinuation compared to those who did not, both in the crude analysis (Crude OR = 0.259; 95% CI: 0.153–0.437; *p* < 0.001) and after adjustment (AOR = 0.424; 95% CI: 0.220–0.819; *p* = 0.011). On the other hand, the use of midwife visits at home was not significantly associated with the breastfeeding outcome (Crude OR = 1.859; 95% CI: 0.535–6.451; *p* = 0.329; AOR = 1.854; 95% CI: 0.506–6.796; *p* = 0.352). Finally, receiving information and assistance from a midwife also showed no statistically significant association with the outcome (Crude OR = 0.753; 95% CI: 0.485–1.169; *p* = 0.206; AOR = 0.847; 95% CI: 0.514–1.397; *p* = 0.516). In summary, concealing during breastfeeding and receiving assistance in breastfeeding were both significantly correlated with reduced odds of discontinuing of exclusive breastfeeding, while midwife home visits and general midwife information/assistance were not significantly related to the outcome.

Table 5 was based on replies to multiple-response questions, where respondents were able to select more than one factor contributing to a reluctance to initiate or early discontinuation of breastfeeding.

The main factors contributing to the reluctance to initiate breastfeeding or its early cessation were identified as: issues with breast health; mental and physical exhaustion; lack of support from medical personnel in mastering effective feeding techniques; and problems related to the baby.

**Figure 2:**
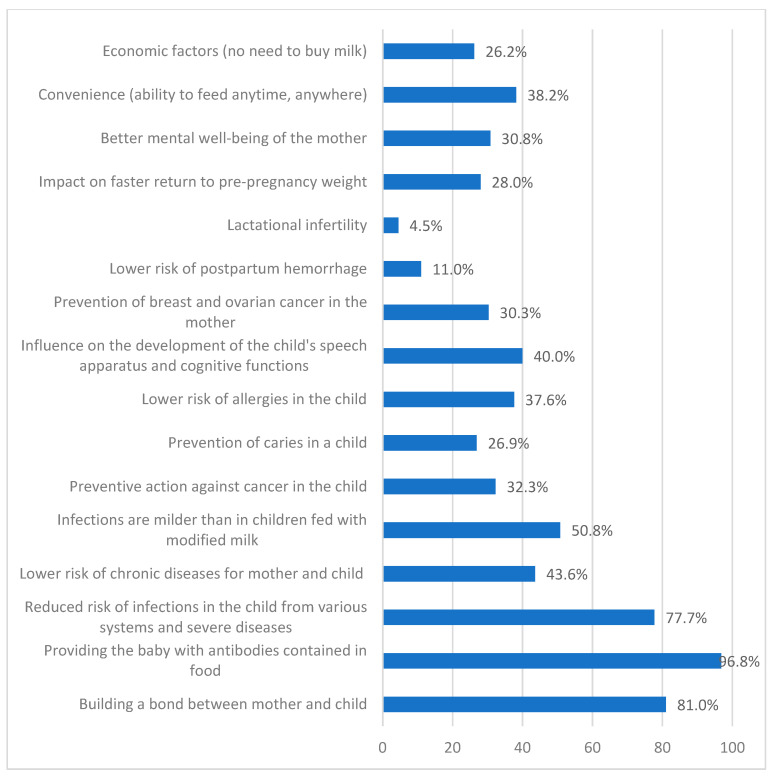
Most important advantages of breastfeeding as perceived by respondents.

In Figure 2, the factors regarding the most important advantages of breastfeeding, based on multiple-response questions, are presented.

The factors identified by respondents as the most significant advantages of breastfeeding include the provision of antibodies in breast milk (96.8%), the establishment of a bond between mother and child (81.0%) and the reduced risk of infections across various systems, as well as the lower likelihood of severe diseases in the child (e.g., necrotising enterocolitis) (77.7%). It is worth emphasising that more than half of the mothers rated the medical support they received as insufficient (65.4%) when reporting breast-related problems (76.1%).

## 4. Discussion

The study was designed to explore the reasons why Polish women discontinue exclusive breastfeeding (EBF) before the infant reaches six months of age, focusing on both barriers and facilitating factors. The authors’ model highlights the central importance of psychological stress and professional midwife support in influencing maternal feeding choices, suggesting that higher stress levels may contribute to earlier formula introduction, while practical breastfeeding support from midwives plays a protective role.

In explaining the mechanism by which stress influences exclusive breastfeeding, it is important to consider that psychological stress can disrupt the release of oxytocin, a hormone that plays a key role in milk ejection during lactation. Continuous impairment of milk ejection can lead to reduced milk production due to incomplete breast-emptying during each feeding. Maternal anxiety, as part of the physiological mechanism, causes increased serum cortisol levels and reduced insulin sensitivity, which result in diminished milk production. This creates a vicious cycle. Maternal anxiety, insufficient milk supply and inadequate weight gain in infants may lead to the decision to introduce formula into the child’s diet. However, the relationship between psychological stress and breastfeeding is bidirectional. The act of breastfeeding reduces maternal stress, likely through its effect on pleasure centres and the calming influence of oxytocin on the mother. The researchers’ observations suggest that interventions to support lactation and breastfeeding in women with high levels of psychological stress would benefit both maternal and child well-being, and that implementing stress-reducing programmes could improve breastfeeding rates [32,33]. Actions supporting EBF could include the widespread use of both screening tests and, when necessary, interventions to reduce maternal stress such as preventing complications and problems during breastfeeding, offering free lactation counselling or providing relaxation classes. This leads to another practical recommendation. Antenatal classes should include content that supports breastfeeding, with the involvement of partners, as they play a significant role in buffering stress and positively influencing the duration of exclusive breastfeeding. The role of support and the need to identify sources of stress are also highlighted by other researchers as key aspects of exclusive breastfeeding. Perceived stress is negatively associated with EBF [34].

Based on the concept of Early life Nutritional Programming (ENP), which emphasises that nutrition during critical periods of development (e.g., infancy), has a lasting impact on metabolism, immune system functioning and the risk of chronic diseases later in life, the promotion of breastfeeding in Poland takes on particular significance [33,35]. Breast milk not only provides ideally balanced nutrients but also contains bioactive molecules (e.g., cytokines, oligosaccharides, IgA and antibodies) that “programme” the infant’s gut microbiome, stimulate the maturation of the intestinal barrier and modulate the immune response, thereby reducing the risk of allergies, obesity and type 2 diabetes [36,37].

It is known that the epidemic of overweightness and obesity greatly affects Polish children and young people: 10% of children aged from one to three are overweight or obese, and 18.4% are at risk of being overweight. The problem also affects almost 30% of eight-year-old children and approximately 20% of children and adolescents aged 10–16. Obesity and type 2 diabetes, which follow, are among the most serious public health challenges in developed countries. Poland is no exception in this respect; thus, efforts should be made, among others, to promote EBF, both for its health benefits and its role in disorder prevention among future generations [38].

Nutrition constitutes the foundation of health and well-being for all, leaving no one behind; it serves as a key element of primary healthcare through promotion and prevention and by addressing its social determinants via people-centred approaches. Healthier populations are achieved through multisectoral actions that extend beyond healthcare systems, although they often leverage the governance, advocacy and regulatory functions of health ministries. Optimal nutrition for individual health and development—exemplified by interventions such as exclusive breastfeeding (EBF)—requires synergy between healthcare systems and broader societal efforts to improve population health [39]. In existing studies, it is confirmed that breast milk can reduce body mass gain in early life, the risk of overweightness/obesity and abdominal obesity, with exclusive breastfeeding (EBF) leading to a 20% slower weight gain compared to formula-fed infants. Evidence indicates that breastfeeding vs. mixed feeding or a longer duration of breastfeeding is associated with lower body mass index (BMI) values, reducing the likelihood of overweightness/obesity in children. This underscores the importance of promoting and sustaining breastfeeding to prevent childhood obesity [40,41]. In a local study from 2018, a model was developed to identify factors associated with almost exclusive breastfeeding beyond six months postpartum. Attendance at antenatal classes, formula supplementation at four months and maternal satisfaction with infant feeding at this time were found to be significant predictors of almost-exclusive breastfeeding in the Polish context. In Poland, antenatal classes represent a widely accessible resource for expectant mothers, offering both theoretical and practical education on pregnancy, childbirth and breastfeeding, in addition to providing current medical guidelines and recommendations [42]. In a subsequent Polish study from 2022, the unsatisfactory rates of breastfeeding in Poland were further underscored. The study, in which maternal attitudes were analysed, demonstrates that, despite recognising the numerous benefits of breastfeeding, many mothers encounter significant barriers to breastfeeding. A frequently reported issue among participants was insufficient care and support from healthcare professionals, which hindered their ability to fully embrace breastfeeding practices. This finding aligns with previous research emphasising the critical role of medical support in promoting successful breastfeeding outcomes [43]. A review by Degla et al. indicated that antenatal education provided by a midwife, breastfeeding counselling and ongoing postnatal support from a midwife, extending up to one year after delivery, are key components of intervention strategies to support and promote breastfeeding. Maternal mental health issues and the increased need for long-term lactation counselling as well as psychotherapy should be carefully considered when designing breastfeeding support interventions [44]. In the present study, it is suggested that stress levels, along with the information and instructional support received during hospital-based perinatal care, are also important drivers of EBF.

The significance of support in breastfeeding is well-documented in international research. Observations by Swerts et al., conducted both at hospitals and primary healthcare facilities, demonstrated that midwives play a crucial role in supporting women during breastfeeding. Their reinforcing and supportive actions contribute to increased maternal confidence and the belief that they are capable of breastfeeding successfully [45]. In a systematic review by McFadden et al., it was confirmed that when midwives provide breastfeeding support, the rates of breastfeeding duration and exclusivity increase [46]. Zhang et al. indicate that mothers perceive adequate breastfeeding knowledge and a positive attitude as key factors for success in maintaining exclusive breastfeeding for the first four months of the child’s life. The authors also highlight that one of the most common reasons for the premature cessation of breastfeeding is the misconception among mothers that the amount of milk is insufficient in the first weeks after birth [47].

Emotional and health values are central to mothers’ perceptions in our study [Figure 2]. This aligns with both local and international findings. Moreover, knowledge about the benefits of breastfeeding, combined with the support received, significantly influences mothers’ decisions regarding infant feeding [48]. Similarly, it is confirmed by Suárez-Cotelo et al. that the level of knowledge about breastfeeding influences both the intention and the type of feeding given to the newborn; thus, this is an element to be taken into account when developing educational strategies to increase breastfeeding rates [49]. Therefore, encouraging mothers-to-be to attend group or individual education does not lose its importance. According to the assumptions of national legislation and financial provisions for health services, all women in Poland have free access to such practices.

Researchers have found that the provision of prelacteal feeds is strongly associated with the delayed initiation of breastfeeding. Self-reported insufficient milk supply remains one of the most common reasons for the introduction of commercial milk formula (CMF) and the cessation of breastfeeding [3]. In the current study, a direct measurement of awareness was not included with regard to the impact of the modified milk industry or the marketing of these products on actual feeding practices, nor were early supplementary feeding and its causes assessed, which could have expanded the interpretation of the authors’ findings.

These results highlight the importance of family support and healthy habits as key determinants of breastfeeding success.

Mothers, despite recognising the many benefits of breastfeeding, encounter certain difficulties in maintaining exclusive breastfeeding such as physical difficulties, emotional stress and insufficient support from medical staff [Table 4]. Insufficient care and support from medical personnel—particularly the limited availability of lactation consultants—has been identified as a commonly reported issue in local research [43]. It is a familiar conclusion that breastfeeding is not only dependent upon a woman’s decisions, but also upon physical and psychological support [1,13]. In response to females indicating a lack of support, compulsory training for medical staff in lactation technique support should be ensured. Free access to lactation counsellors in hospitals and for mothers at home should also be introduced. Currently, individual lactation counselling does not receive government funding in Poland; lactation care is the responsibility of community midwives. In addition, educational campaigns for families, highlighting their role in supporting lactating mothers, would also be valuable. Furthermore, hospitals should be encouraged to use the Baby-Friendly Hospital Initiative (BFHI) standards. Since 1992, the Committee for the Promotion of Breastfeeding has conferred the prestigious title of “Child-Friendly Hospital” to 95 Polish hospitals; currently, only about two-thirds of these maintain a certificate confirming the standard of care for breastfeeding mothers [50].

It is necessary, in the authors’ opinion, to take advantage of the high initiation rates of breastfeeding among Polish women and to ensure support in the following months of the child’s life, which may result in good breastfeeding outcomes among Polish women.

### 4.1. Limitations

This study has several limitations that should be considered when interpreting the findings. First, the cross-sectional design does not allow for causal inference; longitudinal studies are needed to better understand the dynamics between stress, support and breastfeeding outcomes. Second, the use of an online survey may have excluded women with limited Internet access or those less engaged in digital environments, potentially reducing the representativeness of the sample. Additionally, most data were self-reported, which may introduce recall bias or social desirability bias, particularly in relation to sensitive issues such as maternal stress, health behaviours and infant feeding practices. Moreover, the authors recognise that the studied sample included a higher proportion of participants with higher education, which may affect the generalisability of the findings to the broader Polish population. However, recent national data demonstrate a growing number of women in Poland with higher education, making this group more visible in survey-based research [51,52]. Nonetheless, healthcare professionals should ensure inclusive communication strategies that also reach women from diverse backgrounds. Furthermore, the findings are geographically and culturally specific to the Polish context, which may limit their generalisability to other countries or populations with different cultural norms or healthcare systems. Thus, caution should be exercised when extrapolating these results to broader or more diverse contexts. Additionally, certain factors that may influence breastfeeding practices were not fully explored in this study. For instance, while maternal stress was considered, broader aspects of maternal mental health and specific cultural norms were not comprehensively analysed. This omission may have constrained the depth of the current findings. Finally, the study was focused solely on mothers of infants aged up to six months, leaving breastfeeding behaviours beyond this period unexamined. Future research should include broader developmental stages, more diverse populations and longitudinal approaches to validate as well as expand upon these findings.

### 4.2. Further Directions and Implications

The findings of this study have practical implications for multiple stakeholders. Healthcare professionals, particularly midwives and lactation consultants, can use these insights to design more effective, targeted support programmes for mothers at risk of early cessation of exclusive breastfeeding, especially those experiencing elevated stress levels. Policymakers may also benefit from this research by identifying gaps in perinatal care and addressing the need to provide systemic support for breastfeeding women, including stress-reduction programmes and accessible lactation services. Furthermore, antenatal educators can incorporate these findings into their curricula, emphasising the importance of psychological well-being and involving partners in the breastfeeding process. By translating the study outcomes into practice, public health efforts can be more effectively aligned to support and prolong exclusive breastfeeding, contributing to improved maternal and infant health outcomes.

## 5. Conclusions

In this study, the authors highlight insights into the factors associated with exclusive breastfeeding (EBF) practices in Poland, offering both theoretical and practical implications for public health strategies. The key challenges identified include: insufficient medical support; physical and mental exhaustion; lack of professional assistance in mastering effective breastfeeding techniques; and difficulties related to the child. The fact that women recognise the numerous benefits of breastfeeding underscores the importance of education and the promotion of breastfeeding among future mothers.

To address these issues, it is imperative to strengthen lactation support systems by integrating comprehensive lactation counselling services within public healthcare frameworks, enhancing training for medical professionals on breastfeeding techniques and encouraging the adoption of the Baby-Friendly Hospital Initiative (BFHI) standards across healthcare facilities. Moreover, targeted educational campaigns should be developed to promote awareness regarding the benefits of EBF as well as to foster a supportive family and community environment for breastfeeding mothers, taking the emotional state of the mother into account. In future research, the effectiveness of these interventions should also be explored to ensure sustainable improvements in breastfeeding rates.

This study provides insight into exclusive breastfeeding (EBF) practices in Poland.

## Figures and Tables

**Table 1:** Description of variables related to infant feeding practices.

Variable	Description
Feeding method	Exclusive breastfeeding, mixed feeding, formula feeding
Duration of exclusive breastfeeding	Reported in weeks
Frequency of breastfeeding	Number of breastfeeding sessions in 24 h
Frequency of formula feeding	Number of formula feeding sessions in 24 h

**Table 2:** Characteristics of the study group.

Characteristics	No.	%
Respondents	1092	100
**Age**		
<25	102	9.3
26–35	848	77.7
36–45	142	13.0
**Education level**		
Primary education	1	0.1
Secondary education	172	15.8
Higher education (Bachelor’s/Master’s Degree)	900	82.4
Vocational education	19	1.7
**Place of residence**		
Village	252	23.1
City < 50 k inhabitants	181	16.6
City 50–150 k inhabitants	161	14.7
City 150–500 k inhabitants	180	16.5
City > 500 k inhabitants	318	29.1
Marital status		
Single	70	6.4
Marriage/Partnership	1011	92.6
Divorced	11	1.0
**Living with the current partner/child’s father**		
Yes	1079	98.8
No	13	1.2
**Financial situation**		
Very good	336	30.8
Good	528	48.4
Satisfactory	214	19.6
Bad	10	0.9
Very bad	4	0.4
**Professional activity**		
Yes	857	78.5
No	235	21.5
**Housing conditions**		
Very good	598	54.8
Good	424	38.8
Satisfactory	64	5.9
Bad	6	0.5
Very bad	0	0
**Parity**		
1	765	70.1
2	280	25.6
3	40	3.7
4	5	0.5
5 or more	2	0.2
**Counselling for effective breastfeeding**		
Yes	457	41.8
No	635	58.2
**Assistance in proper breastfeeding**		
Yes	521	47.7
No	571	52.3
**Use of midwife visits at home**		
Yes	968	88.6
No	124	11.4
**Receiving information and assistance from midwife**		
Yes	613	56.1
No	479	43.9
**Level of perceived stress**		
Low	235	21.5
Moderate	412	37.7
High	445	40.8

**Table 3:** Logistic regression analysis for factors related to discontinuation of exclusive breastfeeding in the group of women experiencing low or moderate stress.

Variables	Grouping of Variables	Crude OR			Adjusted OR		
		OR	95% CI	*p*	OR	95% CI	*p*
Counselling for effective breastfeeding	No	1.000		0.427	1.000		0.790
	Yes	0.852	0.573–1.265		1.085	0.595–1.976	
Assistance in proper breastfeeding	No	1.000		0.119	1.000		0.369
	Yes	0.731	0.494–1.04		0.760	0.418–1.382	
Use of midwife visits at home	No	1.000		0.856	1.000		0.481
	Yes	1.081	0.466–2.509		1.375	0.567–3.333	
Receiving information and assistance from a midwife	No	1.000		0.125	1.000		0.059
	Yes	0.734	0.494–1.090		0.654	0.421–1.017	

**Table 4:** Logistic regression analysis for factors related to the discontinuation of exclusive breastfeeding in the group of women experiencing high stress.

Variables	Grouping of Variables	Crude OR			Adjusted OR		
		OR	95% CI	*p*	OR	95% CI	*p*
Counselling for effective breastfeeding	No	1.000		<0.001	1.000		0.033
	Yes	0.272	0.157–0.471		0.467	0.232–0.941	
Assistance in proper breastfeeding	No	1.000		<0.001	1.000		0.011
	Yes	0.259	0.153–0.437		0.424	0.220–0.819	
Use of midwife visits at home	No	1.000		0.329	1.000		0.352
	Yes	1.859	0.535–6.451		1.854	0.506–6.796	
Receiving information and assistance from a midwife	No	1.000		0.206	1.000		0.516
	Yes	0.753	0.485–1.169		0.847	0.514–1.397	

**Table 5:** Factors most likely to contribute to a reluctance to initiate or early discontinuation of breastfeeding.

Answers	N	%
Physical exhaustion	700	64.1
Mental exhaustion	793	72.6
Breast health problems (e.g., soreness, nipple injuries, inflammation)	831	76.1
Bust shape variation	136	12.5
Abnormal shape of warts (e.g., flat, concave warts)	327	29.9
Insufficient or no food	584	53.5
Problems on the part of the child (e.g., inability or unwillingness to suckle, drowsiness at the breast)	674	61.7
Lack of assistance from medical personnel in mastering effective feeding techniques	714	65.4
The need to feed “on demand” or to pump regularly	340	31.1
Taking medications related to the mother’s illness	307	28.1
Willingness to return to work quickly	228	20.9
Addictions: nicotinism, alcoholism	206	18.9
Total	5840	-

Multiple-response questions. Percentages do not add up to 100.

## Data Availability

The original contributions presented in the study are included in the article. Further inquiries can be directed to the corresponding author.
